# Annotations

**Published:** 1900-06-09

**Authors:** 


### ANNOTATIONS.

### Vivisection and the Hospitals.

THE frequency with which discussions arise as to the propriety or otherwise of performing experiments upon animals for the sake of advancing our knowledge of the laws of life and of disease, and the acrimony with which this question is too often argued, make it desirable that we should lay down the position held by, as we imagine, the majority of humane and thinking medical men, and probably by the great mass of thoughtful people of all degrees who have not been drawn into the vortex of angry controversy. We hold no brief for those who perform experiments upon animals, and, indeed, our interest in the question arises chiefly from the relation which such experiments have to the prevention and cure of human disease, and feeling as we do that if medicine is to advance, and, if we are ever to obtain greater power over man's ailments than we now possess, those of us who practise medicine must search out the secrets of disease by every means at our disposal, we do not see how we can, or, for that matter, why we should, refuse the advantages offered to us in this regard by experiments made upon animals when carried out under all the guarantees imposed by law upon those who conduct such investigations in this country. Our first duty is the cure of disease, and in view of the trust which is reposed in us, and of the confidence with which patients place their lives in our care (on the understanding surely that we will do all in our power to save them), it seems to us that it would be dishonest in the extreme were we, in deference to any personal dislike to the method, or from any personal shrinking from such experimentation, to withhold from our patients one iota of the advantages which may be gained by experiments carried on in pathological or physiological laboratories. Other people may look upon this matter, if they like, from other points of view. We do not quarrel with them so long as they use truth in argument. It is quite open to them, if they choose so to do, to place animals first, and man second. To those of us, however, who have devoted our lives to the care of the sick, and, so far as in us lies, to the cure of disease, such a position is absolutely impossible. Our duty is to man, and we are bound in honour to bring all knowledge, from whatever source it may be collected, to bear upon the task entrusted to us, to help us in curing man's diseases. If this involves hurting an animal now and again we are sorry, but it cannot be helped. We do not spare ourselves; the nurses who help us do not spare themselves. We and they face danger—do we not sometimes face death?—in order that we may help our patients; and, knowing all this, and recognising how much all who have undertaken the care of the sick are ready to undergo in order that the best may be done for them, when we are asked to stand aside and do something less than our best, and that merely because if we are to do our best the life of a rabbit may be sacrificed in the process, we say that the suggestion is absurd, and that those who make it are mere theoretical triflers, whose opinions are unworthy of consideration by an active and a strenuous world working always to lighten the burden laid upon humanity by injury and disease. This is the reasonable view and must always be the medical view in regard to the admissibility and advisability of such experiments. And as with the doctor so with the hospital. The hospital's first duty is to cure its patients. It is not a mere boarding-house or nursing institute. It is, above all things, a place for the cure of disease, and whatever may be the personal feelings of its supporters in regard to various outside questions, their first duty is to provide the hospital with everything which conduces to the cure of those who entrust themselves to its charity, even though—as a sort of by-product—so doing may involve the connection of one or more of the hospital staff with some institution in which experiments are made upon animals. The first thing is that the patients should get relief.

### The History of a Quarrel.

THE difference between the Obstetrical and the Gynæcological Societies to which we alluded a few weeks ago has led to such an *impasse* that it has been arranged to postpone for the present any decision as to the place of meeting of the Gynæcological Congress of 1902. The trouble began years ago, but the seed of this particular difficulty was laid in 1896, when the Gynæcological Society took upon itself to invite the International Congress of Gynæcology at its meeting at Geneva to meet in London in 1899. This fell through, and the Congress went to Amsterdam. In 1899, however, Dr. Jacobs, of Brussels, the secretary of the Congress, wrote to the secretary of the British Gynæcological Society asking whether the society were prepared to re-issue the invitation for 1902. Nothing loth, the society, which is an energetic body, sent the invitation. In September, 1899, however, *i.e.*, after the Amsterdam Congress, Dr. Engelmann, President of the American Gynæcological Society, who evidently well knew how the London Societies loved one another, urged on the President of the Gynæcological Society the importance of harmonious and concerted action on the part of the two London societies, and, possibly as a way of removing one apple of discord, suggested that Professor Simpson, of Edinburgh, would be a president most acceptable to Americans. The Gynæcological was most happy, and on October 17th, 1899, its President wrote to the President of the Obstetrical, saying that his society was desirous of co-operating with the Obstetrical in inviting and entertaining the next International Congress in 1902. The answer was that the Obstetrical thought it "unadvisable that the two societies should issue a joint

ANNOTATIONS—*continued.*

invitation to the International Congress of Obstetrics and Gynæcology." Nothing daunted, however, the Gynæcological Society set to work to get Professor Simpson as president, and on February 22nd he accepted the invitation. Meanwhile, the officials abroad were evidently not quite happy about the status of the proposed Congress in London. The Americans wanted to go to Barcelona; Dr. Mendes de Leon, of Amsterdam, wanted some official certainty that Dr. Simpson would preside; Dr. Jacobs hoped that the Obstetrical Society would join in order to make this session "as brilliant as the preceding ones"; while Dr. Engelmann put the matter quite definitely as follows: "I believe that I voice the sentiments of those whom I represent when I say that the feeling is general that the invitation could be accepted only, and the Congress be a success only, if the two great societies of Great Britain are united in their desire to see the Congress in their midst, and I sincerely trust that some understanding has been arrived at." Before Dr. Engelmann's letter had been received, however, the invitation had been accepted by the officials of the Congress, and soon afterwards the Council of the Gynæcological Society resolved itself into the nucleus of a committee of organisation, and the circular was issued which led to the boycott, on which we have already commented. The result of this threatened boycott, and of the evidence thereby afforded of the state of tension among the London gynæcologists, has been that Professor Simpson has declined to act as president, and that there is every probability of the whole scheme falling through. Dr. Jacob, writing to the President of the Gynæcological Society on May 28th, says: "On the advice of a number of the founders, I think the proper course is to ask you to postpone any decision till after the meeting of the Congress at Paris on August 2nd." The whole business is much to be regretted. English gynæcologists ought not to be made a laughing stock just because a few foreigners, in accepting an invitation, did not happen to recognise the relative positions occupied by half-a-dozen London specialists. One can understand the Obstetrical Society feeling ruffled at the position seized by the Gynæcological; but its members must have known what was going on, and surely it would have been better to have nipped the thing in the bud, as they could easily have done by the smallest hint, rather than let it run on to such a public washing of dirty linen.

### Investors and Visionaries.

THE New York State Legislature has recently passed a Tenement House Commission Bill, which is exciting a good deal of comment. The City Commissioner of Buildings, Mr. Thomas J. Brady, strongly objects to the appointment of the commission. A committee of the Charity Organisation Society has been inquiring into the housing of the poor in New York City of late, and Mr. Brady does not like them, and thinks this commission will prove another of the same. "I regard that committee," he says, "as a sort of committee of self-appointed busybodies, who undertake to lay down certain rules which should be followed by philanthropic persons, and who attempt to tell charitable millionaires how they ought to spend their money. These committeemen are not practical. They are not builders. They are not investors. They are visionary theorists." A great many persons on this side of the Atlantic are of Mr. Brady's opinion. Yet it may be doubted if it is not they themselves who are the visionaries and the theorists. They see—in a vision, for they will never see it elsewhere—a happy land, where a nation is great and strong and progressive, although its workers live unhealthy and stunted lives in overcrowded homes. They are visionaries, who believe that you can have anæmic men and consumptive women, and yet have no paupers to maintain. They are visionaries, who believe that a nation can compete with its neighbours in trade and industry with workers who live under conditions that make perfect health impossible. That is Mr. Brady's vision. Perhaps the New York State Legislature, taking a larger view, think otherwise. Certainly the charitable millionaires who spend their superfluous money in providing good houses for their workers will never be the less millionaires for that. Good conditions of work, of which good housing is not the least, help to make good workers, and certainly help to keep them when they have been found.

### Drunkenness in the French Army.

IF it be consoling to know that we have companions in misfortune, we British may find some consolation in the fact that the subject of drunkenness is causing great concern to the more thoughtful of our neighbours across the Channel. A French writer, M. Urbain Gohier, in an article published in the *Aurore* on May 6th, declaims against drunkenness as ardently as any of our temperance advocates in this country. Moreover, he considers drunkenness to be a political danger. The voices that shout the loudest in riotous political meetings are those of drunkards; alcoholism is at the bottom of Boulangism, of nationalism, of every war cry that has of late years excited the French nation. The spread of drunkenness in the army is also engaging the attention of the wise. As a consequence of inquiries that have been made on this point, the Minister of War has just addressed a letter to the generals in command of the various army corps. In this document, the Minister (General Gallifet) says that he has considered two alternatives, one of which was simply to restrict the hours during which spirituous liquors might be sold at the regimental canteens, the other the complete abolition of the sale of these, and that he has decided that the latter was the only satisfactory course. "I have decided," he says, "to interdict absolutely the sale in the canteens of all *eau-de-vie*, or spirituous liquor of any kind, as well as the innumerable preparations known as appetizers." The last term is meant to cover absinthe and the various bitters which a digestion weakened by indulgence craves for. The General does not, however, forbid the sale of ordinary fermented liquors, such as wine, beer, cider, perry, and, of course, permits the sale of tea, coffee, chocolate, and the like. It is only spirits that the interdict deals with. Perhaps General Gallifet thinks, as do many people, not altogether foolish, on this side of the Channel, that pure wine and beer are not very unwholesome or dangerous drinks.

